# Analytical Model for the Molecular Ionization Energy in an External Electric Field

**DOI:** 10.1021/acs.jpclett.4c01297

**Published:** 2024-06-04

**Authors:** Per-Olof Åstrand

**Affiliations:** Department of Chemistry, NTNU - Norwegian University of Science and Technology, NO-7481 Trondheim, Norway

## Abstract

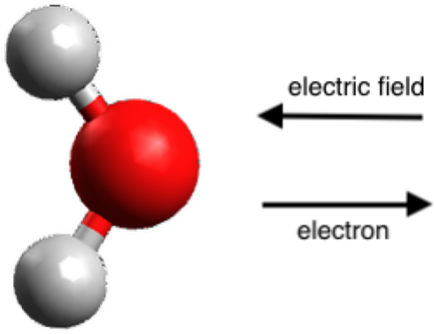

A model for the molecular ionization energy in an applied electric field is presented on the basis of a perturbation expansion in the electric field. The leading term arises from the Frenkel approach, which is the same for all molecules normally used in the Poole–Frenkel model for conductivity in an electric field. For a set of test molecules, the quality of the results is comparable to that of previous results using constrained density functional theory. We conclude that the Frenkel term is dominant and sufficient at relatively low fields and that the dipole and polarizability terms, the leading terms dependent on the properties of the individual molecule, make a significant contribution only at high fields and for relatively large molecules. Because the presented model is analytical, quantum chemical calculations are avoided for a variety of electric field strengths and molecular orientations, and the model can therefore be applied directly in coarse-grained models for electronic processes in dielectric condensed phases.

The ionization energy (ionization potential is an alternative term, but ionization energy is preferred here) of molecular systems in an external electric field is of general interest in electrochemistry. Our interest in modeling this property has mainly focused on molecules of interest for dielectric liquids as electrically insulating materials in, for example, transformers.^1^ We have previously developed a method for the field-dependent ionization energy first based on quantum chemical calculations of the cation interacting with an electron described by a point charge in an electric field.^2,3^ This method was later extended to describe the electron as a spherically symmetric orbital by constrained density functional theory (CDFT),^4^ and the results have been presented for molecules used as base liquids and additives in electrically insulating liquids.^5,6^ As discussed in the original work,^4^ the calculations may be cumbersome because many calculations are needed for each field strength and for each orientation of the molecule in the field. In addition, convergence problems in the CDFT calculations appeared for some molecules and field strengths, leading to the calculations being by no means automatic. A theoretically more elaborate model for one- and two-electron systems has previously been presented.^7^ In addition, related works adopting quantum chemical calculations mainly discussing the dependence of molecular and cation energies or orbital energies and other molecular properties on the electric field have recently been quite extensive.^8−15^

We here suggest a simpler method for calculating the molecular ionization energy in an electric field as compared to the CDFT approach that results in an analytical function that is dependent on the electric field and on molecular properties calculated without an electric field. We expect that the model can be used directly in coarse-grained methods for electronic processes in which the molecular ionization energy is needed.

For the ionization energy, we regard the following chemical reaction for a molecule M
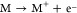
1In the gas phase and without an external electric field, the energy for electron  is zero when it is infinitely far from cation M^+^. Therefore, the ionization energy for this case is calculated as the difference in energy between the cation and the molecule. Here, the system interacts with an external electric field, *E*, and therefore an energy contribution arises from the interaction between the electron and the field. Perturbation and response theory are standard techniques in quantum chemistry for obtaining the properties of a response to an electromagnetic field like molecular polarizabilities.^16−18^ The related approach for the field dependence of excited electronic states is known as the Stark effect,^19^ but a perturbation approach along these lines has not yet been used for the ionization energy in an electric field. If a Taylor expansion in the electric field is adopted, the energy for the product side in the reaction in eq 1, the cation and the electron, , becomes (SI units are used in this work)
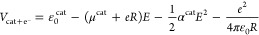
2where ε_0_^cat^, μ^cat^, and α^cat^ are the ground state energy, dipole moment, and polarizability of the unperturbed (without an electric field) cation, respectively, and *e* is the elementary charge. We assume that the dipole moment is aligned with the electric field so that the interaction energy is −*μE*, and therefore the position of the electron, with a charge of −*e*, will be at position −*R* resulting in a dipole moment of *eR* for the electron. The last term is the Coulomb interaction between the cation and the electron.

This energy has a maximum for a certain *R* because it consists of a competition between two terms. The dipole term becomes more important with an increase in *R* because applying the external electric field results in a force in different directions for the positively charged cation and the negatively charged electron, respectively. On the contrary, the Coulomb interaction between the cation and the electron becomes more attractive with a decrease in *R* and attempts to keep the cation and electron together. The maximum of  as a function of *R* is therefore obtained as the balance between the dipole term and the Coulomb term as
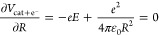
3which results in
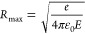
4Because
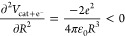
5it is apparent that eq 4 corresponds to a maximum and not a minimum. If the result in eq 4 is included in eq 2, we arrive at
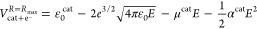
6The term behaving as √*E* is exactly the term derived by Frenkel in his extension of the Poole model for conductivity,^2,20^ which in the literature is normally termed the Poole–Frenkel model for conductivity.^21^ We will here refer to this contribution as the Frenkel term, and it is noted that the Frenkel term is universal in the sense that it does not depend on any molecular properties. The corresponding energy for the molecule, *V*_mol_, is
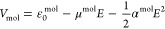
7so that the ionization energy, *V*_IE_, becomes
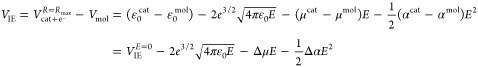
8where *V*_IE_^*E*=0^ is the ionization energy without an applied electric field calculated as the difference between the energy of the cation and the energy of the molecule. Here, we define the differences in the molecular properties between the cation and the molecule as *Δμ* = μ^cat^ – μ^mol^ and *Δα* = α^cat^ – α^mol^, respectively. We note that the equations are dependent on the choice of the origin of the geometry of the cation. In eq 2, it is reflected in the Coulomb term where distance *R* has to be specified between a given but arbitrary point in the cation and the position of the electron, whereas in eq 6, it appears in the dipole moment of the cation, which is dependent on the origin. The dipole term in eq 2 is, however, independent of the choice of origin if the same origin is chosen for the calculation of the dipole moment of the cation and distance *R*.

The ionization energy in eq 8 can also be rewritten as
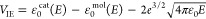
9where the notation implies that quantum chemical calculations are carried out for the cation and the molecule at various external fields. Here, the origin dependence appears in that ε_0_^cat^(*E*) is dependent on the origin when *E* ≠ 0.

In this study, we restrict the field strength to 0–100 MV/cm, which is a range that is considerably larger than those in some of our previous studies of molecules of interest for electrically insulating liquids, where typically electric fields considerably below 20 MV/cm are discussed.^22,23^ The reason is that electrically insulating liquids traditionally are based on essentially nonpolar hydrocarbons, whereas the molecular electric field strength in polar liquids like water is ∼100 MV/cm.^24^ In this context, it is important to note the distinction for condensed phases between the applied field (voltage) and the local electric field experienced by the molecules, for example, modeled by molecular simulations and local field factors.^25,26^ In related work on mass spectrometry, field ionization and thereby electric fields of ≤500 MV/cm have been considered.^27,28^

Let us first analyze the magnitude of the various terms in eq 8 by regarding the water molecule, which has been studied before by us as a model system.^4^ The ionization energy for the water molecule is presented in Figure 1, including only the first two terms in eq 8, i.e., excluding the dipole and polarizability terms. Thus, the only difference between different molecules is the onset on the *y*-axis, i.e., the ionization energy in the gas phase. As reference values for the discussion, the Frenkel term lowers the ionization energy by −4.1 eV at 30 MV/cm and −7.6 eV at 100 MV/cm, which is universal for all molecules within a point charge model. Thus, there is no way that the Frenkel term can be ignored in the calculation of the field-dependent ionization energy by regarding only the field-dependent energies of the molecule and cation in eq 9.

**Figure 1:**
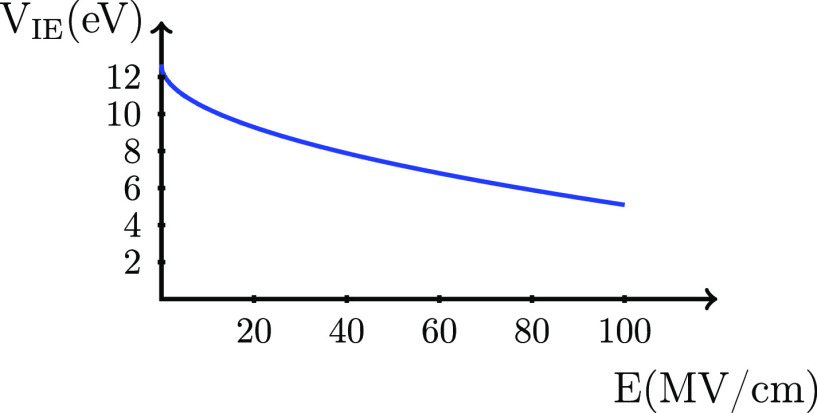
Ionization energy, *V*_IE_, of the water molecule as a function of electric field *E*. The only field-dependent term included in the graph is the Frenkel term, and thus, the graph looks the same for all molecules apart from the ionization energy at *E* = 0.

The ionization energy for a molecule in an external electric field is characterized by distance *R*_max_ given by eq 4 that gives the maximum energy, i.e., the transition state for the ionization process. Distance *R*_max_ is plotted against electric field *E* in Figure 2, and we note that the distance for most cases is considerably longer than the intermolecular distances in condensed phases. Because free electrons are very reactive, it is likely that electron transfer processes between molecules take place rather than a full dissociation process, for example, as has been modeled for polymers.^29,30^

**Figure 2:**
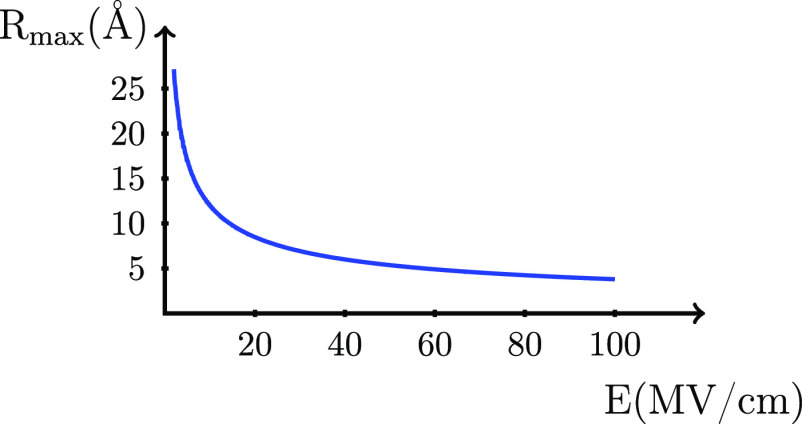
Distance *R*_max_ as a function of electric field *E*.

The contribution in eq 8 from the difference in the dipole moment between the cation and the molecule, *Δμ*, is illustrated in Figure 3. For example, a value of *Δμ* of ∼15 D is required to give a contribution of −1 eV to the ionization energy at an electric field of 30 MV/cm. For the water molecule, the calculated *Δμ* is 0.25 D (μ^mol^ = 1.88 D, and μ^cat^ = 2.13 D), leading to a contribution of −0.016 eV to the ionization energy at 30 MV/cm, which gives a negligible contribution compared to the Frenkel term also at 100 MV/cm because the contribution from the dipole moment is linear in the field.

**Figure 3:**
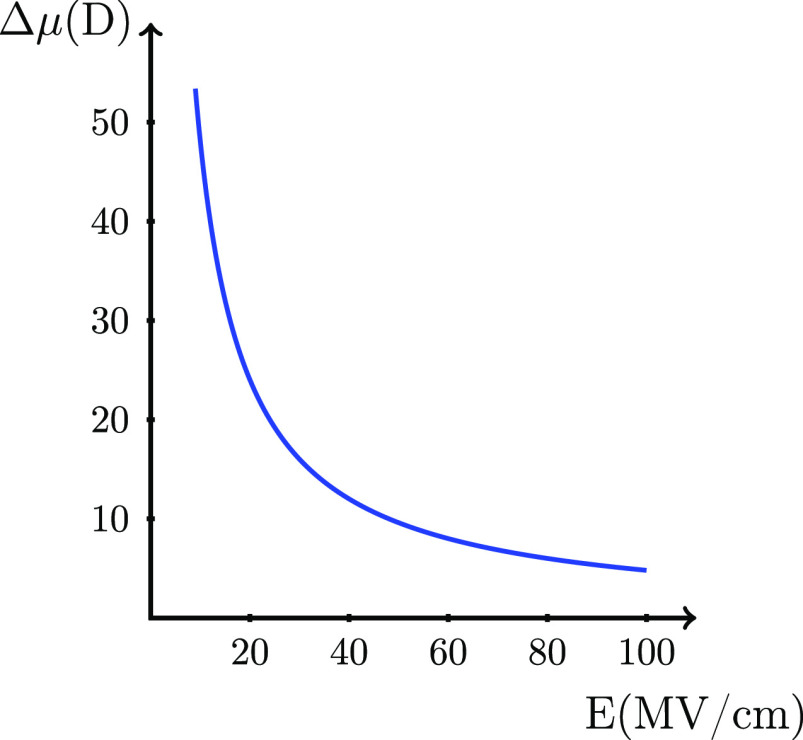
*Δμ* = μ^cat^ – μ^mol^ as a function of *E* to give a contribution to the ionization energy of −1 eV.

In addition, the contribution from the difference in polarizability, *Δα*, in eq 8 is illustrated in the same way in Figure 4. The required *Δα* to give a shift in the ionization energy of 1 eV (assuming that the cation is less polarizable than the molecule) is 320 Å^3^ at 30 MV/cm and 29 Å^3^ at 100 MV/cm. Because the molecular polarizability is ∼1 Å^3^ for the water molecule and the calculated *Δα* becomes −0.64 Å^3^ (α_*zz*_^mol^ = 1.42 Å^3^, and α_*zz*_^cat^ = 0.78 Å^3^), adopting the α_*zz*_ contribution to the polarizability tensor (where the *z*-axis is along the dipole axis), this contribution is negligible for the water molecule. The field-dependent ionization energy of the water molecule therefore seems to be described entirely by the Frenkel term, a result supported by the similarity to the results for water using CDFT.^4^

**Figure 4:**
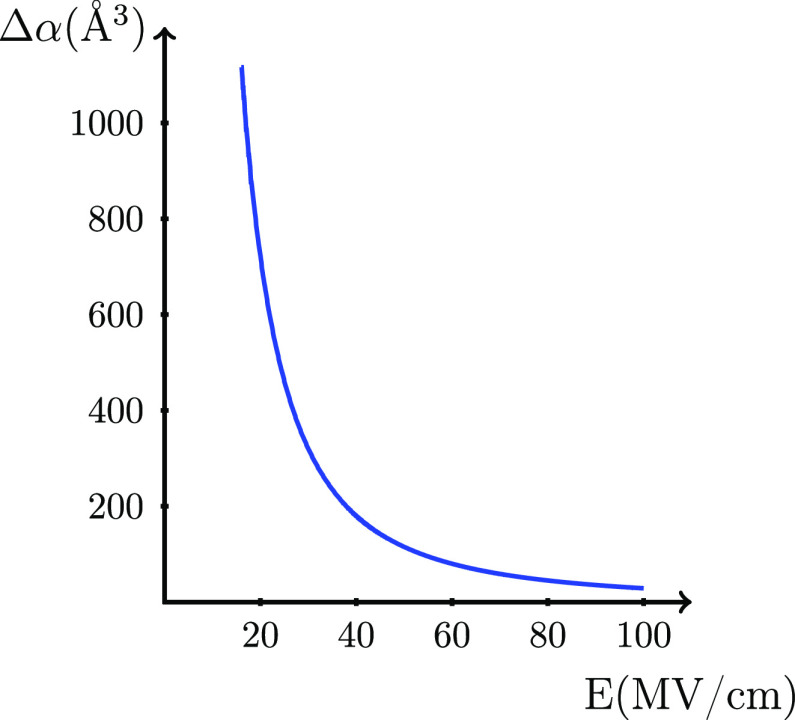
*Δα* = α^cat^ – α^mol^ as a function of *E* to give a contribution to the ionization energy of −1 eV.

Two terms not included in eq 2 are the interactions between the dipole moment and polarizability of the cation with the electron. If we assume that these two terms are small compared to the Coulomb interaction between the charge of the cation and the electron so that the relation for *R*_max_ in eq 4 is still valid, we obtain
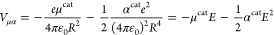
10where we used eq 4 in the second step. Consequently, the last part of this equation is valid only at the *R*_max_. The magnitude of these two contributions can also be evaluated by inspection of Figures 3 and 4 by regarding μ^cat^ instead of *Δμ* and α^cat^ instead of *Δα*, respectively, on the *y*-axis in the graphs. For water, the contributions to the ionization energy from μ^cat^ and α^cat^ at 100 MV/cm are −0.44 and −0.03 eV, respectively, which for μ^cat^ are significant but still small compared to the Frenkel term of −7.6 eV.

The results for three other molecules studied by us before, trichloroethylene^3^ and two unpolar aromatic molecules, benzene^2,5,6^ and pyrene,^6^ are also included. For trichloroethylene, a result similar to that of the water molecule at 100 MV/cm is found for a μ^cat^ of −0.32 eV (μ^mol^ = 0.90 D, and μ^cat^ = 1.51 D), whereas the contribution from α^cat^ is now significant, −0.35 eV (the isotropic polarizability was used: α^mol^ = 10.1 Å^3^, and α^cat^ = 9.9 Å^3^). The contribution from α^cat^ for benzene is −0.39 eV, and for pyrene, it is −1.50 eV at 100 MV/cm (the largest in-plane component of the polarizability was used; for benzene, α^mol^ = 11.9 Å^3^ and α^cat^ = 11.3 Å^3^; for pyrene, α^mol^ = 42.0 Å^3^ and α^cat^ = 43.1 Å^3^). For all molecules apart from pyrene, the contribution from *Δα* is larger than zero; i.e., the polarizabiltiy of the cation is smaller than the polarizability of the corresponding molecule and is thereby not decreasing the ionization energy with an increase in the electric field. This contribution is, however, negligible for all molecules with a contribution of <0.05 eV at 100 MV/cm for all molecules. Because the molecular polarizability increases with the size of the molecular system and to a good approximation is additive,^31^ it is likely that the contribution from α^cat^ in eq 10 is the contribution that in general has to be included first in addition to the Frenkel term, which is supported by the fact that the largest contribution in this work is the contribution for pyrene from the interaction between the cation polarizability and the electron.

To summarize and to generalize eqs 8 and 10 to any orientation of the molecule in relation to the electric field, the molecular ionization energy may be written as
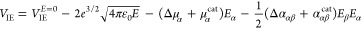
11where the subscripts α and β are the Cartesian coordinates *x*, *y*, or *z* and the Einstein summation convention is adopted. We still assume that the electron leaves the molecule along the direction of the electric field.

In line with these findings, the molecules in our previous work seem to have significant contributions to the field-dependent ionization energy only from the Frenkel term and show similar results, even if the details of the results of each molecule should be investigated in more detail for example by a comparison of eqs 8 and 9. That the Frenkel is dominant is also in line with experimental work,^32−35^ and our original work on field-dependent ionization energies in which a successful parametrization was carried out with a field dependence adopting only a √*E* term.^2^ We should, however, keep in mind that the CDFT approach is also a relatively crude model for the ionization energy.

Different ionization mechanisms in an electric field such as impact ionization, photoionization, and field ionization have been discussed in some detail elsewhere.^2^ The field dependence is much stronger for the ionization energy than for the molecular excitation energies,^3^ as one can understand from the Frenkel term in eq 8, which is dominant for the ionization energy but absent for excitation energies. At relatively high electric fields, we will therefore only have a two-state system: the electronic ground state and the ionized state. When the ionization energy approaches zero, field ionization may appear and may be interpreted as a tunneling effect,^2^ and one can therefore not expect that field ionization can be treated by a perturbation expansion around *E* = 0.

The model presented here may be extended beyond a one-center multipole expansion by adopting atomic charges and distributed polarizabilities in line with force fields for molecular simulations,^36^ which will be required if *R*_max_ is smaller than the relevant intramolecular distances. Considering that only the linear response to the electric field and polarizabilities but not hyperpolarizabilities are normally included when polarizable force fields are used in molecular simulations and that the molecular electric field strengths in polar liquids are ∼100 MV/cm, the truncation of the Taylor expansions in eqs 2 and 7 should be justified, thereby assuming that the contribution from the field dependence of the molecular polarizability is relatively small.

A method for calculating the molecular ionization energy in an electric field is presented on the basis of a perturbation expansion in the electric field, consequently avoiding quantum chemical calculations with a finite electric field. It is found that the Frenkel term is dominant; thus, all molecules have a similar dependence on the electric fiels, and the magnitudes of the leading molecule-specific contributions, the dipole and polarizability terms, are discussed for some model systems. For electric fields, well below the threshold for field ionization and for molecules of modest size, the approach presented here is valid, and in many cases, the universal Frenkel term is the only term needed in a model.

## Details of Calculations

The quantum chemical calculations have been carried out with NWChem version 7.0.2,^37^ utilizing Kohn–Sham density functional theory (DFT) with the CAM-B3LYP functional^38^ and the aug-cc-pVTZ basis set,^39^ which should give reasonable results for a model study. The reason for using a long-range-corrected functional as the CAM-B3LYP functional is that we include calculations of molecular polarizabilities. Because some of the calculations are carried out on cation radicals, unrestricted DFT calculations are utilized for these systems. The geometries have been optimized for both the molecule and the cation; i.e., adiabatic (and not vertical) ionization energies are presented, and thereafter, the dipole moment and the molecular polarizability were calculated. Upon adoption of eq 8, the effect of the electric field on the molecular geometry is not included, but the model may be extended in this direction by considering vibrational polarizabilities.^40^ The dipole moment of a cation is dependent on the origin, and in this work, the center of mass of the molecules has been used as the origin in the calculations.

## References

[ref1] Smalø H. S.; Åstrand P.-O.; Ingebrigtsen S. Calculation of ionization potentials and electron affinities for molecules relevant for streamer initiation and propagation. IEEE Trans. Dielect. Elect. Insul. 2010, 17, 733–741. 10.1109/TDEI.2010.5492245.

[ref2] Smalø H. S.; Hestad Ø.; Ingebrigtsen S.; Åstrand P.-O. Field dependence on the molecular ionization potential and excitation energies compared to conductivity models for insulation materials at high electric fields. J. Appl. Phys. 2011, 109, 073306 10.1063/1.3562139.

[ref3] Davari N.; Åstrand P.-O.; Ingebrigtsen S.; Unge M. Excitation energies and ionization potentials at high electric fields for molecules relevant for electrically insulating liquids. J. Appl. Phys. 2013, 113, 143707 10.1063/1.4800118.

[ref4] Davari N.; Åstrand P.-O.; Van Voorhis T. Field-dependent ionization potential by constrained density functional theory. Mol. Phys. 2013, 111, 1456–1461. 10.1080/00268976.2013.800243.

[ref5] Davari N.; Åstrand P.-O.; Unge M.; Lundgaard L. E.; Linhjell D. Field-dependent molecular ionization and excitation energies: Implications for electrically insulating liquids. AIP Adv. 2014, 4, 037117 10.1063/1.4869311.

[ref6] Davari N.; Åstrand P.-O.; Unge M. Density-functional calculations of field-dependent ionization potentials and excitation energies of aromatic molecules. Chem. Phys. 2015, 447, 22–29. 10.1016/j.chemphys.2014.11.023.

[ref7] Smirnov M. B.; Kraĭnov V. P. Critical fields for ionization of the hydrogen molecule and the molecular hydrogen ion. J. Exp. Theor. Phys. 1997, 85, 447–450. 10.1134/1.558329.

[ref8] Borpuzari M. P.; Boruah A.; Kar R. Ionisation potential theorem in the presence of the electric field: Assessment of range-separated functional in the reproduction of orbital and excitation energies. J. Chem. Phys. 2016, 144, 164113 10.1063/1.4947241. 27131537

[ref9] Tao Z.; Wang X.; Wei Y.; Lv L.; Wu D.; Yang M. A theoretical study of molecular structure, optical properties and bond activation of energetic compounds FOX-7 under intense electric fields. Chem. Phys. 2017, 483–484, 122–131. 10.1016/j.chemphys.2016.12.004.

[ref10] Li J.; Wang Y.; Wang F.; Liang S.; Lin X.; Chen X.; Zhou J. A study on ionization potential and electron trap of vegetable insulating oil related to streamer inception and propagation. Phys. Lett. A 2017, 381, 3732–3738. 10.1016/j.physleta.2017.09.037.

[ref11] Wang Y.; Lin X.; Wang M. Effect of an electric field on the molecular properties of tributyrin, tricaproin and tricaprylin: a theoretical study. J. Korean Phys. Soc. 2021, 79, 369–379. 10.1007/s40042-021-00208-w.

[ref12] Wang Y.; Lin X.; Wang M.; Wang J. A DFT study on the molecular properties of synthetic ester under the electric field. Open Phys. 2021, 19, 647–656. 10.1515/phys-2021-0077.

[ref13] Ye W.; Hao J.; Gao C.; Zhang J.; Zhang J.; Liao R. Discharge mechanism difference analysis between natural ester and mineral oil under impulse electric field: a DFT investigation. IEEE Trans. Dielect. Elect. Insul. 2022, 29, 1803–1810. 10.1109/TDEI.2022.3194494.

[ref14] Zheng H.; Lv W.; Li X.; Feng Y.; Yang E.; Liu C.; Wang Z. Electrical properties of insulating liquids based on molecular properties calculated by density functional theory. IEEE Trans. Dielect. Elect. Insul. 2022, 29, 2274–2282. 10.1109/TDEI.2022.3214618.

[ref15] Wang Y.; Lin X.; Wang M.; Li X. Properties of CF_3_SO_2_F under the influence of external electric field: A DFT study. Results Phys. 2023, 45, 106248 10.1016/j.rinp.2023.106248.

[ref16] Buckingham A. D. Permanent and induced molecular moments and long-range intermolecular forces. Adv. Chem. Phys. 1967, 12, 107–142. 10.1002/9780470143582.ch2.

[ref17] Sauer S. P. A. Molecular Electromagnetism: A Computational Chemistry Approach; Oxford University Press: Oxford, U.K., 2011.

[ref18] Norman P.; Ruud K.; Saue T. Principles and Practices of Molecular Properties: Theory, Modeling, and Simulations; Wiley: Chichester, U.K., 2018.

[ref19] Condon E. U.; Shortley G. H. The Theory of Atomic Spectra; Cambridge University Press: Cambridge, U.K., 1935.

[ref20] Frenkel J. On pre-breakdown phenomena in insulators and electronic semi-conductors. Phys. Rev. 1938, 54, 647–648. 10.1103/PhysRev.54.647.

[ref21] Dissado L. A.; Fothergill J. C. Electrical Degradation and Breakdown in Polymers; Institute of Electric Engineers: Herts, U.K., 1992.

[ref22] Lesaint O. Prebreakdown phenomena in liquids: propagation ’modes’ and basic physical properties. J. Phys. D: Appl. Phys. 2016, 49, 144001 10.1088/0022-3727/49/14/144001.

[ref23] Wang K.; Wang F.; Lou Z.; Han Q.; Zhao Q.; Hu K.; Huang Z.; Li J. Relationship between the electrical characteristics of molecules and fast streamers in ester insulation oil. Int. J. Mol. Sci. 2020, 21, 974 10.3390/ijms21030974. 32024099 PMC7038060

[ref24] Harder E.; Eaves J. D.; Tokmakoff A.; Berne B. J. Polarizable molecules in the vibrational spectroscopy of water. Proc. Natl. Acad. Sci. U.S.A. 2005, 102, 11611–11616. 10.1073/pnas.0505206102. 16081533 PMC1187998

[ref25] Davari N.; Haghdani S.; Åstrand P.-O.; Schatz G. C. Local electric field factors by a combined charge-transfer and point-dipole interaction model. RSC Adv. 2015, 5, 31594–31605. 10.1039/C5RA04183J.

[ref26] Davari N.; Daub C. D.; Åstrand P.-O.; Unge M. Local field factors and dielectric properties of liquid benzene. J. Phys. Chem. B 2015, 119, 11839–11845. 10.1021/acs.jpcb.5b07043. 26241379

[ref27] Basuri P.; Sarkar D.; Paramasivam G.; Pradeep T. Detection of hydrocarbons by laser assisted paper spray ionization mass spectrometry (LAPSI MS). Anal. Chem. 2018, 90, 4663–4668. 10.1021/acs.analchem.7b05213. 29522332

[ref28] Takayama M.; Ubukata M.; Nagatomo K.; Tamura J.; Kubota A. Quantum chemical analysis of molecular and fragment ions produced by field ionization of methyl stearate. J. Am. Soc. Mass Spectrom. 2023, 34, 2731–2738. 10.1021/jasms.3c00277. 37902792

[ref29] Sato M.; Kumada A.; Hidaka K.; Hirano T.; Sato F. Can classical Marcus theory describe hole transfer in polyethylene? IEEE Trans. Dielect. Elect. Insul. 2016, 23, 2978–2984. 10.1109/TDEI.2016.7736861.

[ref30] Unge M.; Aspåker H.; Nilsson F.; Pierre M.; Hedenqvist M. S. Coarse-grained model for prediction of hole mobility in polyethylene. J. Chem. Theory Comput. 2023, 19, 7882–7894. 10.1021/acs.jctc.3c00210. 37842881 PMC10653082

[ref31] Sylvester-Hvid K. O.; Åstrand P.-O.; Ratner M. A.; Mikkelsen K. V. Frequency-dependent molecular polarizability and refractive index: Are substituent contributions additive?. J. Phys. Chem. A 1999, 103, 1818–1821. 10.1021/jp981196g.

[ref32] Duncan M. A.; Dietz T. G.; Smalley R. E. Two-color photoionization of naphthalene and benzene at threshold. J. Chem. Phys. 1981, 75, 2118–2125. 10.1063/1.442315.

[ref33] Chewter L. A.; Sander M.; Müller-Dethlefs K.; Schlag E. W. High resolution zero kinetic energy photoelectron spectroscopy of benzene and determination of the ionization potential. J. Chem. Phys. 1987, 86, 4737–4744. 10.1063/1.452694.

[ref34] Douin S.; Parneix P.; Brechignac P. Solvent shift of the ionization potential of the aniline-argon system. Z. Phys. D 1991, 21, 343–348. 10.1007/BF01438407.

[ref35] Erdmann N.; Nunnemann M.; Eberhardt K.; Herrmann G.; Huber G.; Köhler S.; Kratz J. V.; Passler G.; Peterson J. R.; Trautmann N.; et al. Determination of the first ionization potential of nine actinide elements by resonance ionization mass spectroscopy (RIMS). J. Alloys Compd. 1998, 271–273, 837–840. 10.1016/S0925-8388(98)00229-1.

[ref36] Engkvist O.; Åstrand P.-O.; Karlström G. Accurate intermolecular potentials obtained from molecular wave functions: Bridging the gap between quantum chemistry and molecular simulations. Chem. Rev. 2000, 100, 4087–4108. 10.1021/cr9900477. 11749341

[ref37] Aprà E.; Bylaska E. J.; de Jong W. A.; Govind N.; Kowalski K.; Straatsma T. P.; Valiev M.; van Dam H. J. J.; Alexeev Y.; Anchell J.; et al. NWChem: Past, present, and future. J. Chem. Phys. 2020, 152, 184102 10.1063/5.0004997. 32414274

[ref38] Yanai T.; Tew D. P.; Handy N. C. A new hybrid exchange-correlation functional using the Coulomb-attenuating method (CAM-B3LYP). Chem. Phys. Lett. 2004, 393, 51–57. 10.1016/j.cplett.2004.06.011.

[ref39] Kendall R. A.; Dunning T. H. Jr.; Harrison R. J. Electron affinities of the first-row atoms revisited. Systematic basis sets and wave functions. J. Chem. Phys. 1992, 96, 6796–6806. 10.1063/1.462569.

[ref40] Bishop D. M. Molecular vibrational and rotational motion in static and dynamic electric fields. Rev. Mod. Phys. 1990, 62, 343–374. 10.1103/RevModPhys.62.343.

